# Gene transfer of human neuregulin-1 attenuates ventricular remodeling in diabetic cardiomyopathy rats

**DOI:** 10.3892/etm.2013.1273

**Published:** 2013-08-27

**Authors:** BINGONG LI, JIAN XIAO, YONG LI, JIAN ZHANG, MINGHUI ZENG

**Affiliations:** Department of Cardiology, First Affiliated Hospital, Nanchang University, Nanchang, Jiangxi 330006, P.R. China

**Keywords:** diabetes mellitus, cardiomyopathy, ventricular remodeling

## Abstract

Neuregulin-1 (NRG-1) is a cardioactive growth factor released from endothelial cells. However, the effect of NRG-1 on ventricular remodeling in diabetic cardiomyopathy (DCM) remains unclear. The aim of the present study was to investigate the pathophysiological role of NRG-1 in a rat model of DCM. Rat cardiac microvascular endothelial cells (CMECs) were transfected with human NRG-1 (hNRG-1) lentivirus. The hNRG-1 medium was utilized to culture rat cardiomyocytes. The cardiomyocytes were counted with a hemacytometer to determine the proliferation index and Annexin V/propidium iodide double staining was employed to examine the apoptotic rate. A rat model of DCM was induced by an intraperitoneal injection of streptozotocin. The hNRG-1 lentivirus was injected into the myocardium of the DCM model rats. Four weeks after the lentiviral injection, cardiac catheterization was performed to evaluate the cardiac function. Apoptotic cells were determined by terminal deoxynucleotidyl transferase-mediated dUTP nick-end labeling (TUNEL) staining. Left ventricular sections were stained with Masson’s trichrome to investigate the myocardial collagen content. The expression levels of related genes and proteins were analyzed. The results indicated that hNRG-1 conditioned medium stimulated the proliferation and counteracted the apoptosis of cardiomyocytes *in vitro*. In the rats with DCM, gene transfer of hNRG-1 to the myocardium improved heart function, as indicated by invasive hemodynamic measurements. In addition, hNRG-1 reduced the number of apoptotic cells, decreased the expression of bax and increased the expression of bcl-2 in the myocardium of the DCM model rats. Myocardial fibrosis and type I and III pro-collagen mRNA levels in the myocardium were significantly reduced by hNRG-1. hNRG-1 also increased the expression of phospho-Akt and phospho-eNOS in the myocardium. In conclusion, the gene transfer of hNRG-1 ameliorates cardiac dysfunction in diabetes. Although further studies are required, NRG-1 appears to protect cardiomyocytes against apoptosis and to reduce the extent of myocardial interstitial fibrosis.

## Introduction

Coronary artery disease and hypertension account for the majority of the myocardial abnormalities that occur in diabetes. However, previous studies have shown that diabetes mellitus alters cardiac structure and function independently of coronary artery disease and systemic hypertension, a condition known as diabetic cardiomyopathy (DCM) (1,2). DCM is characterized by systolic and diastolic dysfunction due to reduced contractility, prolonged relaxation and decreased compliance of the myocardium (3,4). The pathological mechanism of DCM is considered to involve myocardial apoptosis and necrosis, reactive hypertrophy, myocardial fibrosis, endothelial dysfunction, disturbance of the management of the metabolic cardiovascular load and cardiac autonomic neuropathy (4–6). Although the features of DCM are well-identified, the pathogenesis underlying the myocardial remodeling process has not been elucidated, and no effective treatment strategy is available.

Neuregulin-1 (NRG-1), a cardioactive growth factor released from endothelial cells, is indispensable for cardiac development and the structural maintenance and functional integrity of the heart (7,8). In the adult heart, NRG-1 expression appears to be restricted to endothelial cells near cardiomyocytes (in the endocardium and in the myocardial microvasculature), while it is absent from larger coronary arteries, veins and the aorta (9). An increasing number of studies have focused on NRG-1 and members of the ErbB family that serve as receptors for NRG-1, in order to better understand the role of this signaling pathway in the physiology and pathophysiology of the heart. Based on studies of isolated cell systems, a number of processes appear to be regulated by NRG-1/ErbB signaling, including cell growth, myofilament structure and organization, survival, myocyte-matrix coupling, glucose uptake and angiogenesis (10–12).

Studies using recombinant human neuregulin-1 (rhNRG-1) containing the epidermal growth factor (EGF)-like domain (necessary for ErbB2/ErbB4 activation) have shown that NRG-1 plays an important role in heart performance (13,14). Therefore, we hypothesized that the gene transfer of NRG-1 would attenuate ventricular remodeling and improve cardiac function through regulating cardiac apoptosis and fibrosis. To test this hypothesis, we investigated the pathophysiological role of NRG-1 in a rat model of DCM induced by streptozotocin (STZ).

## Materials and methods

### Animals

Male Sprague Dawley (SD) rats were obtained from the Animal Center of Nanchang University (Nanchang, China). The experiments were performed in compliance with the ARRIVE Guidelines on Animal Research (15). All the procedures were approved by the Institutional Animal Care and Use Committee of Nanchang University.

### Preparation of rat cardiac microvascular endothelial cells (CMECs)

Neonatal SD rats (70–80 g) were anesthetized and the hearts were removed and retrogradely perfused with Dulbecco’s modified Eagle’s medium (DMEM; Gibco, Carlsbad, CA, USA) for 5 min through the ascending aorta to remove blood cells. Following the removal of connective tissue, the remaining left ventricles were separately finely minced and digested with 0.2% collagenase I (Sigma, St. Louis, MO, USA) in Hank’s balanced salt solution for 30 min at 37°C in a shaking water bath. Trypsin (0.02%; Gibco) was then added and the mixture was incubated for an additional 5 min. The digested solution was filtered through an 100-*μ*m mesh filter. The filtrate was collected and the cells were plated on culture dishes coated with human fibronectin (Invitrogen, Carlsbad, CA, USA) and maintained in DMEM supplemented with 20% fetal bovine serum (FBS; HyClone, Logan, UT, USA), human vascular endothelial growth factor (Invitrogen), human fibro-blast growth factor (Invitrogen) and human EGF (Invitrogen). After a 3-day culture, unattached cells were removed and fresh medium was added to the adherent cells. The medium was replaced once every 3 days. The cells were cultured to 80% confluence before being released with EDTA (Sigma) and subcultured. Some of the adherent cells at passage 3 were collected for immunofluorescence analyses, which was performed by staining using factor VIII (Solarbio, Beijing, China).

### Lentiviral construction and gene transfer

A recombinant lentivirus containing human NRG-1 (pLV-hNRG-1) was constructed. Briefly, a full-length hNRG-1 gene cDNA was cloned into the lentivirus shuttle plasmid vector pGC-FU, which contains a cytomegalovirus promoter and a polyadenylation signal of bovine growth hormone. For the construction of lentivirus containing green fluorescent protein (GFP), a shuttle vector containing human phosphoglycerate kinase gene promoter was used. The control virus lacking the hNGR-1 gene was separately prepared. Recombinant lentivirus was generated by homologous recombination and propagated in 293T cells (Genechem, Shanghai, China). At 48 h after transduction, the supernatant from the 293T cells was collected and purified by cesium chloride density gradient centrifugation and stored in 10 mmol/l Tris-HCl (pH 7.4), 1 mmol/l MgCl_2_, and 10% (v/v) glycerol at −70°C. Virus titers were determined by a plaque assay on 293T cell monolayers.

For transduction, CMECs at passage 3 were plated at a density of 1×10^5^/well into 6-well dishes with DMEM/20% FBS. The cells at each well were incubated with 20 MOI NRG-1 lentivirus (NRG-1-CMEC) or 20 MOI GFP lentivirus (GFP-CMEC) for 72 h. Lentiviral infection was validated by visualization of enhanced GFP under a fluorescence microscope (Nikon, Tokyo, Japan). Subsequently, the medium was replaced with fresh DMEM/20% FBS and the cells were cultured for an additional 48 h. At the end of the incubation period, the media and cells in each well were collected and analyzed.

### ELISA for cytokines in CMEC media

The levels of hNRG-1 in the media were analyzed by enzyme immunoassay using a human neuregulin-1 ELISA kit according to the manufacturer’s instructions (PlantSelect Biotechnology Systems Ltd., Dartmouth, NS, Canada). Data were expressed as the mean ± SEM.

### Cardiomyocyte culture

Rat hearts were surgically removed from 1- to 3-day-old SD rats, washed instantly with phosphate-buffered saline (PBS) solution, and then minced into 1- to 3-mm^3^ pieces. The minced tissue was subjected to 6–8 cycles of proteolytic dissociation by magnetic stirring (10 min, 37°C) in 0.06% trypsin solution. The supernatants from each cycle were pooled and centrifuged. The cell pellet was resuspended in DMEM supplemented with 20% FBS. Selective adhesion was achieved by incubation at 37°C for 1.5 h in a humidified atmosphere (5% CO_2_ and 95% air) in order to obtain a high purity of cardiomyocytes. Subsequently, 0.1 mM bromodeoxyuridine (Sigma) was added to the medium for the first 48 h of culture to inhibit the growth of fibroblasts.

### Assessment of the bioactivity of conditioned CMEC media

Cardiomyocytes were plated into 12-well dishes at a cell density of 2×10^4^/well and randomly allocated into three groups. The cells in group A were cultured in fresh DMEM/20% FBS (control); the cells in group B were cultured in 50% fresh DMEM/20% FBS and 50% the medium previously harvested from GFP-CMECs; and the cells in group C were cultured in 50% fresh DMEM/20% FBS and 50% the medium previously harvested from hNRG-1-CMECs. After 3 days of culture, some wells of cardiomyocytes in each group were counted. The number of cardiomyocytes was independently determined by investigators blinded to the type of cell culture using a hemacytometer.

The remaining wells of cardiomyocytes of the three groups were incubated with 20 ng/ml tumor necrosis factor-α (TNF-α) (Peprotech, Rocky Hill, NJ, USA). The cardiomyocytes were harvested 24 h later, stained with Annexin V-APC/propidium iodide (PI; KeyGen Biotech, Nanjing, China) and subjected to flow cytometric analysis to assess apoptosis following the manufacturer’s instructions.

### Animals and lentivirus injection

SD rats at a postnatal age of 6 weeks (body weight, 200–220 g) were allocated to the control (n=8) and diabetic groups (n=30). Diabetes was induced by the intraperitoneal (i.p.) injection of STZ (50 mg/kg; Sigma, L’Isle d’Abeaux, France) (16). Tail vein blood glucose was measured every 3 days during the first week; the rats with plasma glucose levels ≥16.7 mmol/l were considered to be diabetic. Concurrently, control rats were injected i.p. with 1 ml/kg body weight 20 mmol/l citrate buffer (pH 4.5) vehicle. The control and diabetic rats both raised on standard food and water for the whole experimental period. Twelve weeks after the induction of diabetes, 24 diabetic rats were used for further analysis; the remaining six rats died or were excluded due to unsuccessful induction of diabetes. The 24 diabetic rats were randomly allocated into three groups: the hNRG-1, GFP and DCM groups (n=8 rats per group). Subsequently, all the diabetic rats were anesthetized with 4% chloral hydrate solution (1 ml/100 g) by i.p. injection and put on an animal ventilator. Thoracotomy was performed. Approximately 50 *μ*l/heart (5×10^7^ TU/ml) of hNRG-1-lentivirus (hNRG-1 group) or GFP-lentivirus (GFP group) or an equivalent volume of PBS alone (DCM group) was injected at five sites in the left ventricles of the rats using a 30-gauge needle. Following the injection of lentiviral vectors, the rats continued to be raised on standard food and water for 4 weeks. All analyses were performed 16 weeks after the induction of diabetes.

### Analysis of myocardial function

To evaluate the cardiac function, cardiac catheterization was performed as previously described (17). Briefly, after the induction of light general anesthesia [4% chloral hydrate solution (1 ml/100 g) by i.p. injection], a catheter was inserted into the right carotid artery and advanced into the left ventricle. Ventricular pressure signals were measured with a transducer and conditioner (MLT0830; ADInstruments, Bella Vista, Australia) and digitally recorded with a data acquisition system (PowerLab; ADInstruments). The following indices were obtained: heart rate (HR), left ventricular systolic pressure (LVSP), left ventricular enddiastolic pressure (LVEDP), as well as the maximum rates of left ventricular pressure rise and fall (+dp/dt max and −dp/dt max, respectively). During this process, the animals were placed on controlled heating pads. Core temperature was measured via a rectal probe and was maintained at 37°C. The rats were sacrificed after analysis of myocardial function and the hearts were harvested for subsequent experiments.

### Histopathological process and detection of apoptotic cells by terminal deoxynucleotidyl transferase-mediated dUTP nick-end labeling (TUNEL) staining

The samples were fixed in 10% formalin and were paraffin-embedded in the Surgical Pathology Facility of Nanchang University. TUNEL analysis was performed with a commercially available kit (Dead End Colorimetric TUNEL System) according to the manufacturer’s instructions (Promega, Madison, WI, USA). The slides were counterstained with hematoxylin (blue). Three midventricular sections (from the apex to the base) of each heart tissue were analyzed. Cardiomyocyte nuclei were quantified by randomly counting 10 fields/section. The apoptotic index (percentage of apoptotic nuclei) was calculated as apoptotic nuclei/total nuclei counted × 100.

### Analysis of myocardial collagen content

Sections of left ventricles were stained with Masson’s trichrome to measure interstitial fibrosis. Interstitial collagen was quantified at a final magnification of ×200 using a microscope (BX51; Olympus, Center Valley, PA, USA) connected to a video camera (DS-Fi1; Nikon, Tokyo, Japan). The images captured under the microscope were used to calculate the collagen volume fraction of the myocardial interstitium using a Computer Imaging Analysis System (MPIAS-500; Nikon, Tokyo, Japan). The content of interstitial collagen, which was expressed as the fractional area of the entire cross-section where the perivascular collagen was excluded, was averaged on nine fields selected across the wall thickness in the septum and free wall.

### Gene expression analysis by quantitative reverse transcription polymerase chain reaction (qPCR)

Tissue samples obtained from the left ventricular free wall were minced. Total RNA was extracted from the samples with TRIzol reagent according to the manufacture’s instructions (Invitrogen). For qPCR, cDNA was synthesized in a 20-*μ*l reaction volume containing 4 *μ*g total RNA and SuperScript™ II Reverse Transcriptase (Fermentas, Burlington, ON, Canada) according to the manufacturer’s instructions. qPCR was carried out with a 7500 Real-Time PCR system (Applied Biosystems, Carlsbad, CA, USA) using SYBR-Green I (Applied Biosystems) as a fluorescent dye according to the manufacturer’s instructions. Relative quantitation of the mRNA expression of the gene of interest was calculated using the comparative threshold cycle number for each sample. To control the variation in the amount of DNA, gene expression of the target sequence was normalized in relation to the expression of an internal control, β-actin. The PCR products of hNRG-1 were size-fractioned by electrophoresis on 2% agarose gels. Primers for hNRG-1, bcl-2, bax, collagen type I and III, as well as β-actin were the following: hNRG-1, forward: 5′-TCACCATGGTGGCGACCGGTTCAGGCAGAGACAGAAAG-3′ and reverse: 5′-TCACCATGGTGGCGACCGGTTCAGGCAGAGACAGAAAG-3′; bcl-2, forward: 5′-CGGGAGATCGTGATGAAGT-3′ and reverse: 5′-CCACCGAACTCAAAGAAGG-3′; bax, forward: 5′-GCAGGGAGGATGGCTGGGGAGA-3′ and reverse: 5′-TCCAGACAAGCAGCCGCTCACG-3′; collagen type I, forward: 5′-GTTCGTGGTTCTCAGGGTAG-3′ and reverse: 5′-TTGTCGTAGCAGGGTTCTTT-3′; collagen type III, forward: 5′-TGCCCACAGCCTTCTACACCCT-3′ and reverse: 5′-CAGCCATTCCTCCCACTCCAG-3′; β-actin, forward: 5′-TGTGCTATGTTGCCCTAGACTTC-3′ and reverse: 5′-CGGACTCATCGTACTCCTGCT-3′.

### Western blot analysis

Samples of left ventricle myocardium were homogenized in tissue protein extraction reagent (Beyotime, Beijing, China) supplemented with protease inhibitors. After centrifugation at 12,000 × g for 10 min at 4°C, the supernatants were collected according to the manufacturer’s instructions. Protein concentration was measured using the BCA Protein Assay kit (Pierce Biotechnology, Rockford, IL, USA). Equal amounts of 20 *μ*g protein were loaded onto 10% sodium dodecyl sulfate-polyacrylamide gels. hNRG-1, total Akt, phospho-Akt (p-Akt; phospho-Ser 473), total eNOS and phospho-eNOS (p-eNOS; phospho-Ser 1177) were detected with mouse anti-hNRG-1, rabbit anti-Akt-1/-2/-3, rabbit anti-phospho-Akt-1/-2/-3 (Ser 473), mouse anti-eNOS and rabbit anti-phospho-eNOS antibodies (Santa Cruz Biotechnology, Inc., Santa Cruz, CA, USA). After probing with these antibodies, the membranes were stripped of bound immunoglobulins. The immunoblots were developed by the enhanced chemiluminofluorescence method (Thermo Fisher Scientific, Hudson, NH, USA). The signals were quantified by densitometric analysis using a chemiluminescence imaging system (General Electric Company, Fairfield, CT, USA) and normalized to those of β-actin, an endogenous control protein.

### Statistical analysis

All the data were expressed as the mean ± SEM. Comparisons between groups were made using one-way analysis of variance (ANOVA) with Fisher’s protected least significant difference post hoc comparison test. P<0.05 was considered to indicate a statistically significant difference.

## Results

### Effects of CMEC supernatants on cardiomyocyte proliferation and survival

CMECs were characterized by positive staining for factor VIII endothelial marker. The cells at passage 3 were transfected with hNGR-1-lentivirus or GFP-lentivirus and the expression of GFP was observed by fluorescence analysis.

Cytokines in the supernatant were measured by ELISA. The level of hNRG-1 in the group transfected with hNGR-1-lentivirus was detected to be 18±5.3 ng/ml, while no hNRG-1 was detected in the group transfected with GFP-lentivirus.

**Figure 1.**
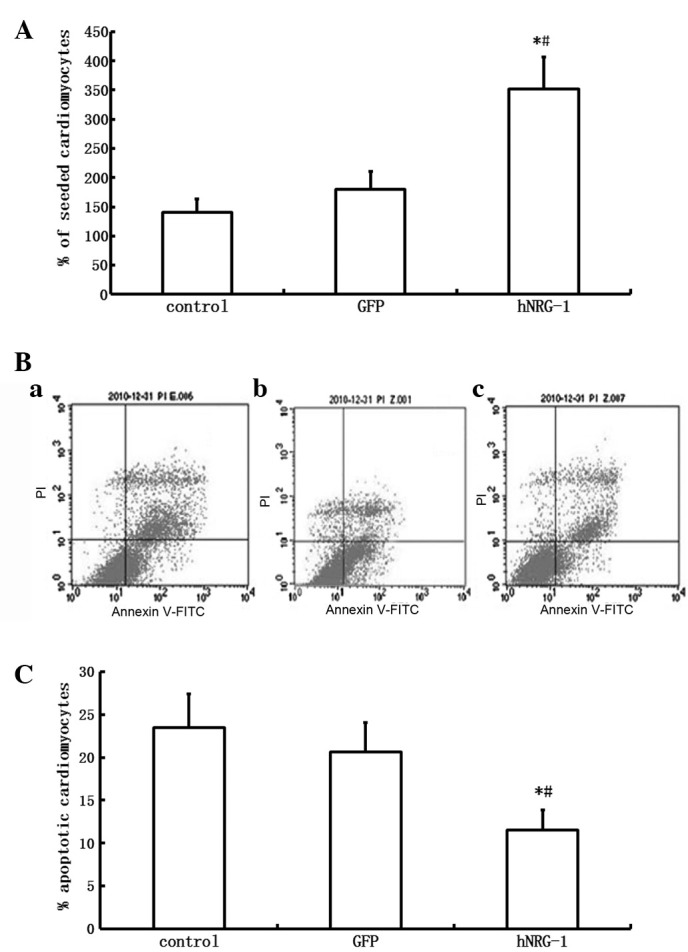
Assessment of the bioactivity of conditioned CMEC media. (A) Effects of conditioned medium on cardiomyocyte growth. Data are expressed as the percentage of cells on day 3 vs. the initial cell number on day 0 (mean ± SEM). The number of cardiomyocytes exposed to hNRG-1 conditioned medium was significantly increased compared with the number of cardiomyocytes exposed to GFP conditioned medium or DMEM/20% FBS medium only (control). (B) Effects of conditioned medium on cardiomyocyte apoptosis induced by TNF-α. Cardiomyocytes were stained with APC-conjugated Annexin V and propidium iodide (PI) and then analyzed by flow cytometry. The cells were stained with APC-Annexin V and PI to analyze apoptotic (Annexin V^+^/PI^−^) cell fractions using flow cytometry. (a) control group; (b) GFP group; (c) hNRG-1 group. (C) After 24 h of induction of apoptosis, the percentage of apoptotic cardiomyocytes exposed to hNRG-1 conditioned medium was lower compared with the percentage of cardiomyocytes exposed to GFP conditioned medium or DMEM/20% FBS medium (control). ^*^P<0.05 vs. control group; ^#^P<0.05 vs. GFP group (n=8 rats per group). CMEC, cardiac microvascular endothelial cell; hNRG-1, human neuregulin-1; GFP, green fluorescent protein; DMEM, Dulbecco’s modified Eagle’s medium; FBS, fetal bovine serum; TNF-α, tumor necrosis factor-α.

Since the proliferation and survival of cardiomyocytes is an important aspect of DCM, the effects of CMEC-conditioned media on cardiomyocyte growth and survival were determined. The number of cardiomyocytes cultured for 3 days in hNRG-1-CMEC conditioned basal medium was significantly higher compared with the number of cardiomyocytes cultured in GFP-CMEC conditioned basal medium (P<0.05). No significant difference was detected in the number of cardiomyocytes between the control and GFP groups (P>0.05, Fig. 1).

After co-culture with TNF-α for 24 h, the apoptotic rates of cardiomyocytes cultured in hNRG-1-CMEC conditioned basal medium was significantly lower compared with the number of cardiomyocytes cultured in GFP-CMEC conditioned basal medium (P<0.05). No significant difference was detected in the apoptotic rates of cardiomyocytes between the control and GFP groups (P>0.05, Fig. 1).

### hNRG-1 expression in cardiac tissues

**Figure 2.**
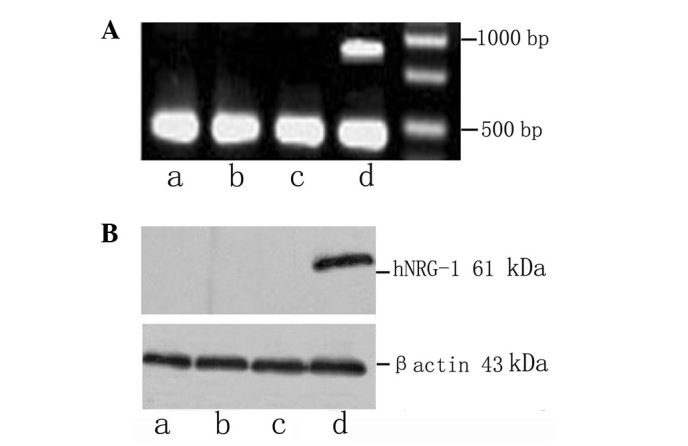
Expression of hNRG-1 in cardiac tissues. (A) hNRG-1 mRNA and (B) the fusion protein of hNRG-1 and GFP were detected in the cardiac samples extracted from the cardiac tissues of the rats in the hNRG-1 group; however, they were not detected in those from the control, DCM and GFP groups. Lane a, control group; lane b, DCM group; lane c, GFP group; land d, hNRG-1 group. hNRG-1, human neuregulin-1; GFP, green fluorescent protein; DCM, diabetic cardiomyopathy.

The expression of hNRG-1 in heart tissues was confirmed by relative quantification of hNRG-1 mRNA and western blot analysis. hNRG-1 mRNA and the fusion proteins of hNRG-1 and GFP were detected in the cardiac samples extracted from the cardiac tissues of the rats in the hNRG-1 group but not in those from the rats in the control, DCM and GFP groups (Fig. 2).

### NRG-1 improves myocardial function

Sixteen weeks after the induction of diabetes, cardiac function was evaluated by invasive hemodynamic measurements. No significant difference was detected in HR among the four groups (P>0.05). A lower LVSP and higher LVEDP were observed in the DCM group compared with those in the control group (P<0.05). Resting maximum rates of rise (+dp/dt max) and fall (−dp/dt max) in left ventricular pressure were also impaired after the induction of diabetes (P<0.05), indicating that systolic and diastolic functions were significantly impaired in the diabetic rats. Following the injection of hNRG-1-lentivirus, these hemodynamic abnormalities were markedly attenuated (P<0.05). However, the hemodynamic abnormalities in the GFP group were comparable with those in the DCM group (P>0.05, Table I).

### NRG-1 protects cardiomyocytes against apoptosis

**Figure 3.**
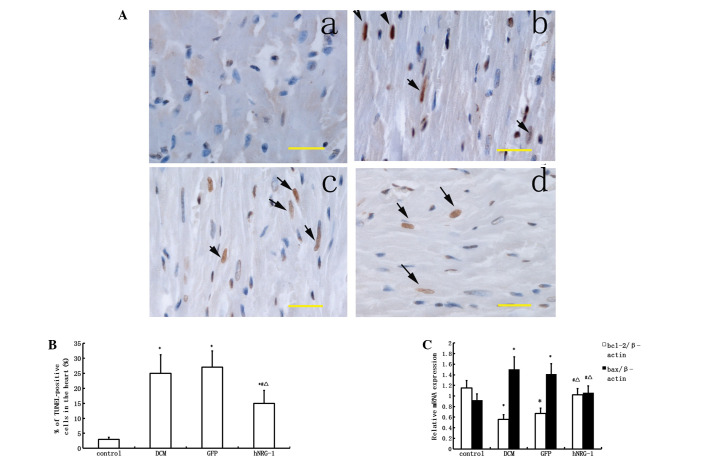
Analysis of cardiomyocyte apoptosis. Cardiomyocyte apoptosis was detected using a TUNEL assay. (A) Representative images following TUNEL staining. Brown-stained cells were TUNEL-positive and considered to be apoptotic. (a) Control group; (b) DCM group; (c) GFP group; (d) hNRG-1 group. Magnification, ×400; scale bar, 100 *μ*m. (B) Statistical analysis of cardiomyocyte apoptosis. The number of TUNEL-positive cells was increased in the cardiac samples from diabetic rats, and decreased following transfection with hNRG-1-lentivirus. (C) Statistical analysis of the expression of apoptosis-related mRNA using PCR. Bax mRNA expression was increased while Bcl-2 mRNA expression was decreased in the DCM group compared with those in the control group. Transfection with hNRG-1-lentivirus resulted in increased Bcl-2 mRNA expression and decreased Bax mRNA expression compared with those in the DCM and GFP groups. ^*^P<0.05 vs. control group; ^#^P<0.05 vs. DCM group; ^Δ^P< 0.05 vs. GFP group (n=8 rats per group). DCM, diabetic cardiomyopathy; GFP, green fluorescent protein; hNRG-1, human neuregulin-1; TUNEL; terminal deoxynucleotidyl transferase-mediated dUTP nick-end labeling; PCR, polymerase chain reaction.

A TUNEL assay was performed to assess apoptosis *in vivo*. The number of positively stained cells in the DCM group was higher than that in the control group (P<0.05). Transfection with hNRG-1-lentivirus significantly reduced the number of apoptotic cells compared with those in the GFP and DCM groups (P<0.05, Fig. 3). qPCR was used to determine the mRNA expression levels of bcl-2 and bax, which are known to be markers of apoptosis. Following qPCR, bcl-2 was shown to be downregulated and bax was upregulated in the DCM group, while these alterations were attenuated following transfection with hNRG-1-lentivirus (P<0.05). Since bcl-2 is known to be an anti-apoptotic and bax a pro-apoptotic protein, these results indicated that transfection with hNRG-1-lentivirus protects cardiomyocytes against apoptosis (Fig. 3).

### NRG-1 attenuates myocardial interstitial fibrosis

**Figure 4.**
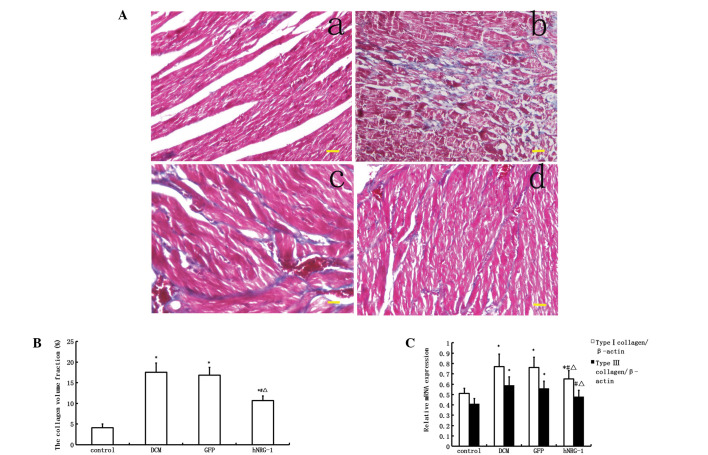
Analysis of interstitial fibrosis. (A) Fibrotic infiltration in the myocardium shown by Masson’s trichrome staining. Blue-stained areas represent fibrotic infiltration. (a) Control group; (b) DCM group; (c) GFP group; (d) hNRG-1 group. Magnification, ×200; scale bar, 100 *μ*m. (B) Quantitative analysis of fibrosis. Collagen volume fraction was higher in the DCM and GFP groups than in the control group; this increased collagen volume fraction was attenuated following transfection with hNRG-1-lentivirus. (C) Statistical analysis of the expression of collagen type I and III mRNA using qPCR. The mRNA expression of collagen type I and III was increased in the DCM and GFP groups compared with the control group; this increased mRNA expression of collagen type I and III was suppressed following transfection with hNRG-1-lentivirus. ^*^P<0.05 vs. control group; ^#^P<0.05 vs. DCM group; ^Δ^P<0.05 vs. GFP group (n=8 rats per group). DCM, diabetic cardiomyopathy; GFP, green fluorescent protein; hNRG-1, human neuregulin-1.; qPCR, quantitative polymerase chain reaction.

The collagen volume fraction, which is an indicator of interstitial fibrosis, was higher in the DCM group than in the control group (P<0.05, Fig. 4). Similarly, the collagen type I and III mRNA expression levels were also significantly upregulated in the DCM group compared with those in the control group (P<0.05, Fig. 4). The levels of myocardial fibrosis and of type I and III pro-collagen mRNA in the myocardium were markedly inhibited following cardiac transfection with rhNRG-1 rather than GFP, suggesting that hNRG-1 treatment attenuates the myocardial interstitial fibrosis caused by diabetes.

### NRG-1 increases the expression of phospho-Akt and phospho-eNOS

**Figure 5.**
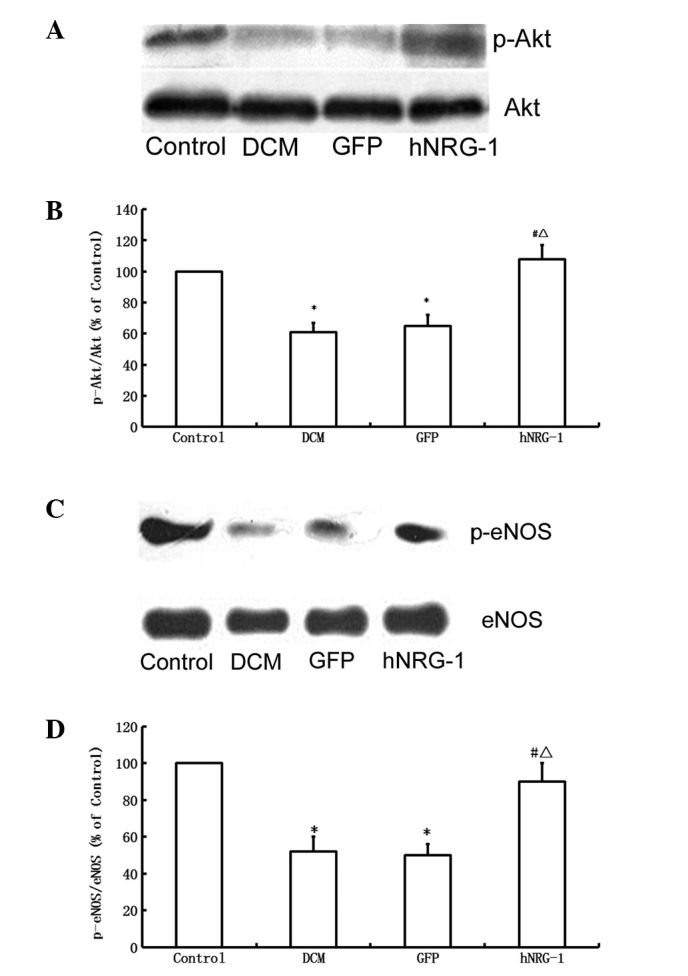
Effects of hNRG-1 gene delivery on Akt and eNOS levels. (A) Western blot and (B) quantitative analyses of phosphorylated (p-Akt) and total Akt levels. (C) Western blot and (D) quantitative analyses of phosphorylated (p-eNOS) and total eNOS levels. ^*^P<0.05 vs. control group; ^#^P<0.05 vs. DCM group; ^Δ^P<0.05 vs. GFP group (n=8 rats per group). DCM, diabetic cardiomyopathy; GFP, green fluorescent protein; hNRG-1, human neuregulin-1.

Western blot analysis showed that STZ treatment reduced the level of Akt phosphorylation compared with that in the control group, while hNRG-1 gene transfer increased the level of phospho-Akt (Fig. 5). Total Akt levels were not altered among the four groups. Similarly, hNRG-1 gene delivery significantly increased the level of the phosphorylated form of eNOS compared with the levels in the GFP and control groups. Total eNOS levels remained unaltered (Fig. 5). These results indicate that hNRG-1 gene transfer resulted in the activation of Akt and eNOS pathways by phosphorylation.

## Discussion

The present study demonstrated that gene transfer of hNRG-1 attenuated the remodeling of the hearts of DCM model rats by regulating cardiomyocyte apoptosis and cardiac fibrosis, in association with enhancement of systolic and diastolic cardiac function. These findings support the hypothesis that NRG-1 plays an important role in the regulation of heart function.

NRG-1 acts as a paracrine factor via the ErbB family of tyrosine kinase receptors expressed in cardiomyocytes. ErbB receptors are a family of four transmembrane receptors that bind multiple growth factors including epidermal growth factor (EGF), transforming growth factor-α (TGF-α) and NRG1-4. ErbB3 is expressed in prenatal myocytes, while adult ventricular myocytes express only ErbB1, ErbB2 and ErbB4. ErbB1, also known as the EGF receptor, does not bind NRG-1; thus, only ErbB2 and ErbB4 serve as NRG-1 receptors in adult cardiomyocytes (18,19). NRG-1 acts through ErbB2 and ErbB4 in a paracrine fashion to stimulate MEK/ERK, Akt/PI3-kinase, Src/FAK and NO synthase, which act synergistically to promote myocyte function and survival in the setting of cardiac stress (20–23).

Increased apoptosis has been shown to play a critical role in the development of DCM (24,25). In the present study, the overexpression of hNRG-1 inhibited cardiomyocyte apoptosis *in vivo* and *in vitro*. The mechanisms of apoptosis inhibition are suggested to be associated with PI3-kinase/Akt pathway, one of the downstream signaling pathways of NRG-1/ErbB. Previous studies have shown that the PI3-kinase/Akt pathway is involved in the protection of cardiomyocytes against cell death, as well as in the regulation of metabolism and growth (26,27). The exact mechanism by which NRG-1-dependent Akt signaling protects myocytes has not been fully elucidated. An Akt-dependent change in bcl-2 family expression has been implicated (28,29). It has been reported that NRG-1 is involved in the regulation of bcl-2 and p-Bad expression through the PI3K/Akt pathway following transient focal cerebral ischemia (30). The results of the present study suggest that the role of NRG-1 in cardiac protection is conferred through activation of the Akt pathway, which is associated with an increase in the level of bcl-2 expression and reduction in the level of bax expression. The ratio of bax/bcl-2 is known to be an important marker of cardiomyocyte apoptosis (31,32).

DCM is considered to involve interstitial and perivascular fibrosis; fibrosis is one of the most important characteristics of DCM (25). Myocardial fibrosis is known to cause myocardial dysfunction in diabetes. Our findings demonstrated that NGR-1 attenuates heart fibrosis. However, the exact underlying mechanism remains unclear. NO synthase, which may be stimulated by the NRG-1/ErbB signaling pathway, is suggested to be involved in myocardial fibrosis. NO synthase has been reported to attenuate myocardial fibrosis by regulating renin release (33). Upregulation of the renin-angiotensin system (RAS) has been described in diabetes and is associated with the development of cardiac hypertrophy and fibrosis. In addition, cardiomyocytes and endothelial cells in the hearts of individuals with diabetes and end-stage heart failure provide evidence of oxidative stress, apoptosis and necrosis that correlate with RAS activation (2). In the present study, certain actions of NRG-1 relating to the inhibition of cardiac remodeling are suggested to depend on the regulation of renin release through NO synthase.

Other mechanisms may be involved in the cardiac protective effect of NRG-1. Evidence indicates that increased oxidative stress contributes to the development and progression of DCM. The activation of RAS has been shown to be associated with increased oxidative damage and cardiomyocyte apoptosis in the diabetic heart, leading to cardiac fibrosis (34–36). Previous studies have shown that NRG-1β treatment of myocytes *in vitro* alters the expression of a number of genes related to regulation of cellular oxidative stress, such as catalase and superoxide dismutase (SOD), which are significantly upregulated following treatment with NRG-1β, supporting the idea that NRG-1β is important in the regulation of myocardial oxidative stress (12). Based on these findings, NRG-1 is postulated to inhibit cardiac remodeling at least partially through the regulation of myocardial oxidative stress. Cardiac autonomic neuropathy is an additional important mechanism of DCM. Previous studies have shown that neuregulin proteins have the ability to control excessive β-adrenergic activation. NRG-1 may induce counterbalancing parasympathetic activity in animal models, which is potentially an additional factor in the protective role of neuregulin in heart failure (37,38).

It has been reported that NRG-1/ErbB expression declined at a later stage of pump failure (39). The decline in NRG-1 expression coincided with the development of eccentric ventricular hypertrophy and pump failure, and was accompanied by a downregulation of the ErbB2 and ErbB4 mRNA levels. This was suggested to be due to the increased levels of angiotensin II and epinephrine, both of which reduce NRG-1 mRNA synthesis in the cardiac endothelium (40). The replenishment of NRG-1 is suggested to inhibit the physiopathological aggravation of DCM.

In conclusion, the gene transfer of hNRG-1 improves cardiac dysfunction in diabetes. Although further studies are needed, NRG-1 appears to be able to protect cardiomyocytes against apoptosis and to reduce the extent of myocardial interstitial fibrosis. Replenishing NRG-1 is suggesting to be an alternative option for DCM treatment, although the exact mechanism of action required investigation in future studies.

## Figures and Tables

**Table I.** Hemodynamic parameters evaluated by invasive measurements.

Group	HR (beats/min)	LVSP (mmHg)	LVEDP (mmHg)	+dp/dt (mmHg/sec)	−dp/dt (mmHg/sec)
Control (n=8)	340±35	135±15	2.3±0.9	6451±408	5819±328
DCM (n=8)	320±31	103±7^a^	11.8±3.2^a^	4901±341^a^	3856±275^a^
GFP (n=8)	318±21	108±11^a^	10.1±2.7^a^	4887±322^a^	3845±259^a^
hNRG-1 (n=8)	329±29	120±12^b,c^	6.4±2.3^a–c^	5908±361^a–c^	4890±311^a–c^

Data are expressed as the mean ± SEM.

a P<0.05 vs. control group;

b P<0.05 vs. DCM group;

c P<0.05 vs. GFP group (n=8 rats per group).

HR, heart rate; LVSP, left ventricular systolic pressure; LVEDP, left ventricular end-diastolic pressure; +dp/dt, maximum rate of left ventricular pressure rise; −dp/dt, maximum rate of left ventricular pressure fall; DCM, diabetic cardiomyopathy; GFP, green fluorescent protein; hNRG-1, human neuregulin-1.
